# Supplementary material for: Cloud BioLinux: pre-configured and on-demand bioinformatics computing for the genomics community
Source: BMC Bioinformatics. 2012 Mar 19;13:42. doi: 10.1186/1471-2105-13-42 (PMC3372431; doi:10.1186/1471-2105-13-42)
Supplement: Additional file 1 — Supplementary 1 Cloud BioLinux software documentation in the form of a mini, self-contained website. Users need to download and uncompress the .zip file, and open through a web browser the "index.html" file available on the main directory. (ZIP 1823 kb). [file 1471-2105-13-42-S1.ZIP › Cloud-BioLinux-Package-Documentation/docs/pamp.html]

Bio-Linux Software Documentation Pages

Back to search form

## pamp

|  |  |
| --- | --- |
| Name | pamp |
| Description | **pamp** is a part of the PAML package, which is a suite of programs for phylogenetic analyses of DNA or protein sequences using maximum likelihood (ML).  **pamp** carries out parsimony-based analyses for a given tree topology, estimation of the substitution pattern by the method of Yang and Kumar (1996)  Most programs in the PAML package have control files that specify the names of the sequence data file, the tree structure file, and models and options for the analysis. The default control files are baseml.ctl for baseml and basemlg, codeml.ctl for codeml, pamp.ctl for pamp, mcmctree.ctl for mcmctree. The progam evolver does not have a control file, and uses a simple user interface. All you do is to type evolver and then choose the options. For other programs, you should prepare a sequence data file and a tree structure file, and modify the appropriate control files before running the programs.  **References:**  Adachi, J., and M. Hasegawa. 1996a. MOLPHY Version 2.3: Programs for molecular phylogenetics based o­n maximum likelihood. Computer science monographs, 28:1-150. Institute of Statistical Mathematics, Tokyo.    Adachi, J., and M. Hasegawa. 1996b. Model of amino acid substitution in proteins encoded by mitochondrial DNA. Journal of Molecular Evolution 42:459-468.[Entrez]    Brown, W. M., E. M. Prager, A. Wang, and A. C. Wilson. 1982. Mitochondrial DNA sequences of primates, tempo and mode of evolution. Journal of Molecular Evolution 18:225-239.[Entrez] |
| Homepage | http://abacus.gene.ucl.ac.uk |
| Remote Documentation | http://abacus.gene.ucl.ac.uk/software/pamlDOC.pdf |
